# Case Report: A novel *IKBKB* variant (c.1705G>T) is associated with immune dysregulation and disseminated tuberculosis

**DOI:** 10.3389/fimmu.2025.1541899

**Published:** 2025-03-07

**Authors:** Gabriel Emmanuel Arce-Estrada, Miguel Rodríguez-Morales, Selma Cecilia Scheffler-Mendoza, Marimar Sáez-de-Ocariz, Laura Berrón-Ruiz, Sara Elva Espinosa-Padilla, Francisco Alberto Contreras-Verduzco

**Affiliations:** ^1^ Unidad de Investigación en Inmunodeficiencias, Instituto Nacional de Pediatría, Mexico City, Mexico; ^2^ Departamento de Genética Humana, Instituto Nacional de Pediatría, Mexico City, Mexico; ^3^ Facultad de Medicina of the Universida Nacional Autónoma de México, Mexico City, Mexico; ^4^ Servicio de Inmunología, Instituto Nacional de Pediatría, Mexico City, Mexico; ^5^ Servicio de Dermatología, Instituto Nacional de Pediatría, Mexico City, Mexico

**Keywords:** *IKBKB*, disseminated tuberculosis, splenic abscesses, acral skin nodules, hemophagocytic syndrome

## Abstract

**Objective:**

To describe a novel *IKBKB* variant linked to immune dysregulation and disseminated tuberculosis, alongside a review of pathogenic variants to outline their phenotypic spectrum.

**Material and methods:**

Observational case report and literature review.

**Results:**

A five-month-old girl from an endogamous Mexican population developed symptoms suggestive of Kawasaki disease which progressed to hemophagocytic syndrome. *Mycobacterium bovis* was found in her skin, blood, and bone marrow. She had received the Bacillus Calmette-Guérin (BCG) vaccine on the second day of life. Genetic testing revealed a homozygous pathogenic variant (PV) in the *IKBKB* gene (c.1705G>T, p.Glu569*). Both parents were heterozygous. Fourteen publications were found, encompassing 33 patients with 14 different PV, including the case described in this work.

**Discussion:**

Hypogammaglobulinemia, candidiasis and mycobacterial infections were common in most cases identified. Our case is unique in presenting with Kawasaki disease, hemophagocytic syndrome, and mycobacteria from skin, blood, and bone marrow.

**Conclusions:**

We identified a novel homozygous PV in the *IKBKB* gene, highlighting new clinical manifestations.

## Highlights:

- An infant girl received the BCG vaccine at birth.
- Kawasaki disease and hemophagocytic syndrome were the initial manifestations.
- *Mycobacterium bovis* was isolated from her skin, blood, and bone marrow.
- A homozygous PV in the *IKBKB* gene was found: c.1705G>T (p.Glu569*).
- 33 cases with 14 PV of the gene were found in a literature review.

## 1 Introduction

The gene *IKBKB* encodes the protein IKKβ, which activates members of the nuclear factor kappa B (NF-κB) transcription factor family through the classical (or canonical) pathway. This activation occurs via the phosphorylation of IκB inhibitors (1, 2). In non-activated cells, NF-kB dimers are associated with molecules of the IkB protein family, which inhibit NF-κB binding to deoxyribonucleic acid (DNA) (1). PV in the *IKBKB* gene can result in severe or milder forms of combined immunodeficiency (3). This condition may be inherited in both autosomal dominant (immunodeficiency type 15A, Online Mendelian Inheritance in Man (OMIM) #618204) or autosomal recessive (immunodeficiency type 15B, OMIM #615592) patterns.

## 2 Materials and methods

### 2.1 Observational case report

We present a previously unreported PV of a 15B immunodeficiency and its associated phenotypic spectrum.

### 2.2 Literature review

A literature search was conducted for the development of this review in the Human Mutation Database, MEDLINE, EMBASE and LILACS databases using the terms “*IKBKB* gene” OR “*IKBKB*”, with emphasis on clinical cases, case series and reviews.

## 3 Results

### 3.1 Observational case report

We describe a five-month-old girl from an endogamous Mexican population who was admitted to our hospital for persistent high fever over a week, malaise and a leukocytosis (29.0 x10^3^/µL) in a complete blood cell test. She had received the BCG vaccine on the second day of life. Her weight and height were adequate for her age and she had no history of infections.

**Figure 1:**
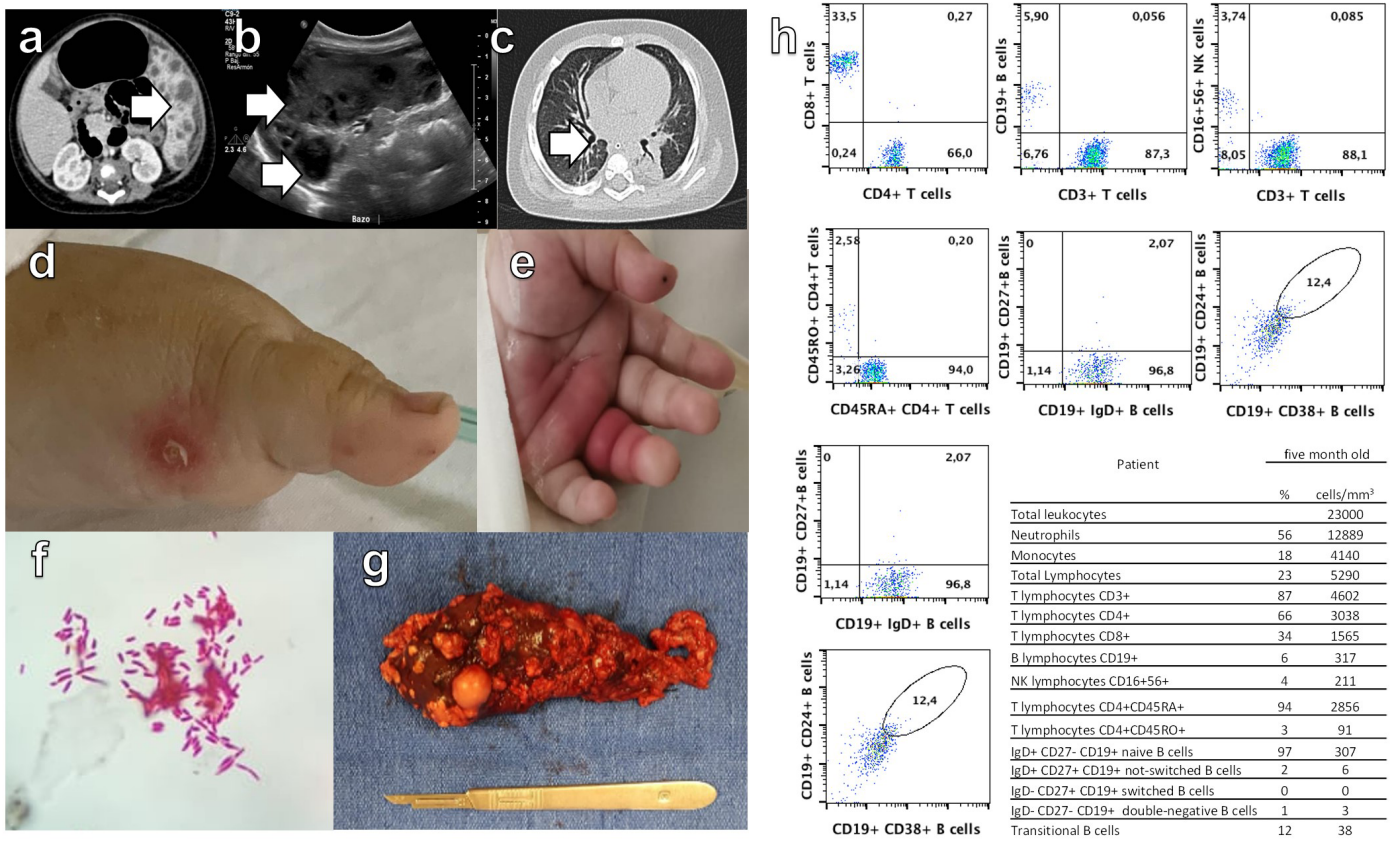
Abdominal computed tomography (CT) scan **(a)** and ultrasound **(b)**, with white arrows indicating multiple abscesses. Image **(c)** shows a pulmonary nodule detected by CT scan. Images **(d, e)** display acral skin nodules. In **(f)**, *Mycobacterium bovis* identification in blood culture is shown. Image **(g)** presents the macroscopic visualization of the spleen removed by surgery. Flow cytometry **(h)** reveals low levels of double-negative T cells, as well as decreased memory B cells without isotype switching and an absence of isotype-switched memory B cells.

The patient was initially diagnosed with incomplete Kawasaki disease, this diagnosis was supported by the presence of high and prolonged fever (over a week), strawberry tongue, angular cheilitis, perineal erythema, BCG vaccination site erythema and induration, anemia, leukocytosis and sterile pyuria, with elevated inflammatory markers such as C reactive protein and pro-brain natriuretic peptide. Echocardiographic findings of pericardial effusion further strengthened this diagnosis and she was treated with intravenous gamma globulin. Also, she presented abdominal pain and elevated serum levels of procalcitonin, D-dimer and ferritin. Imaging studies revealed findings consistent with splenic abscesses and a nodule in the right lung. (**Figures 1A–C**).

Subsequently, her clinical course evolved with features consistent with hemophagocytic lymphohistiocytosis (HLH). The patient fulfilled more than five diagnostic criteria, including fever, splenomegaly, neutropenia, thrombocytopenia, hypertriglyceridemia, hypofibrinogenemia, hyperferritinemia and confirmed hemophagocytosis in 2 bone marrow biopsies.

She was treated with intravenous steroids, cyclosporine and gamma globulin. The bone marrow biopsy also identified bacilliform microorganisms, so an inborn error of immunity was suspected. Immunoglobulin levels were not measured prior to the administration of intravenous gamma globulin. However, post-infusion levels were reported as follows: IgG 968 mg/dL (normal range: 215-704 mg/dL), IgA 5 mg/dL (normal range: 8.1-68 mg/dL), IgM 25 mg/dL (normal range: 35-102 mg/dL), and IgE 0.2 KUA/L.

She developed acral skin nodules with superinfection of some (**Figures 1D, E**) and the microscopic analysis revealed bacillary structures embedded in the cytoplasm of macrophages. *Mycobacterium bovis* was isolated from blood culture, bone marrow and the acral skin nodules biopsy (**Figure 1F**). Human Immunodeficiency Virus and leishmaniasis were excluded and the patient was treated for tuberculosis with isoniazid, rifampin, pyrazinamide, and ethambutol. There was no improvement in the febrile pattern until the spleen was surgically removed (**Figure 1G**). Histopathological examination revealed multiple splenic abscesses associated with bacillary colony infection with abundant macrophages.

Once the fever has subsided, flow cytometry was performed showing low levels of double negative T cells as well as low memory B cells without isotype switching and no isotype-switched memory B cells (**Figure 1H**).

**Figure 2:**
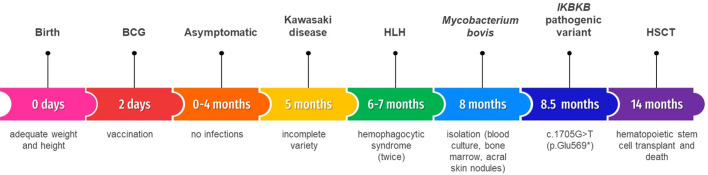
Timeline.

Continuing with the diagnostic protocol and after informed consent, a massive parallel sequencing with the most current version of Invitae´s (Invitae Corp. San Francisco California, U.S.) primary immunodeficiencies panel available (407 genes) was requested. It reported a homozygous pathogenic variant in the *IKBKB* (NM_001556.2) gene: c.1705G>T (p.Glu569*).The extension study showed that parents are heterozygous. She received an haploidentical hematopoietic stem cell transplant (HSCT) with conditioning based on antithymocyte globulin, fludarabine and busulfan. She died ten days later because of septic shock. The timeline is shown in **Figure 2**.

### 3.2 Literature review

**Figure 3:**
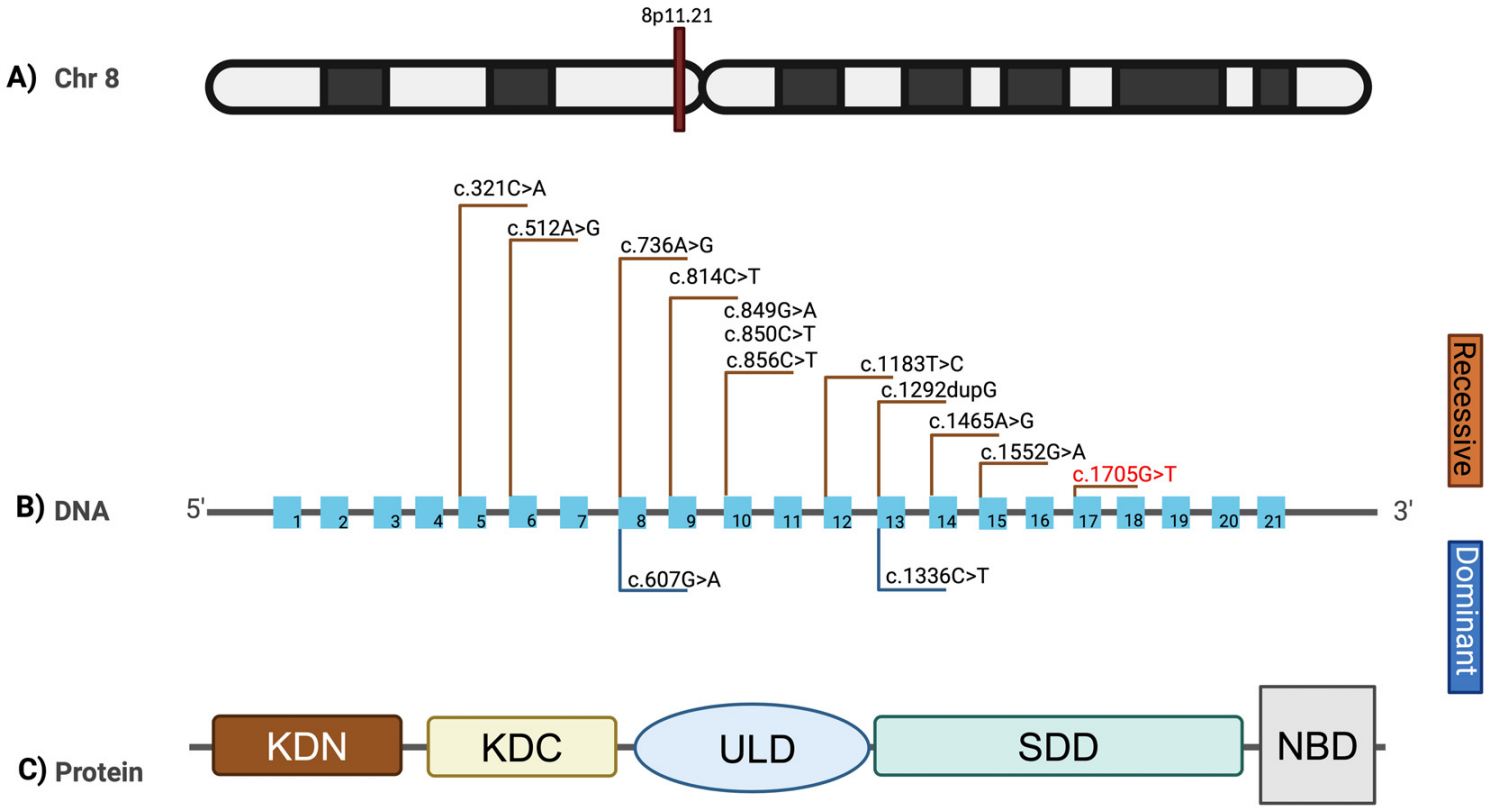
Mutational spectrum of the *IKBKB* gene. **(A)** Ideogram representing the 8p11.21 locus where the *IKBKB* gene is located. **(B)** Mutational spectrum of the variants reported in the *IKBKB* gene. The variants described with autosomal recessive inheritance are shown above, and the variant corresponding to this work is shown in red. The variants reported in a heterozygous state with an autosomal dominant inheritance pattern are shown below. **(C)** Structural representation of the domains of the *IKBKB* protein. N-terminal kinase domain (KDN), C-terminal kinase domain (KDC), ubiquitin-like domain (ULD), scaffold dimerization domain (SDD), and NEMO-binding domain (NBD). Created in BioRender. Contreras, **(f)** (2025) https://BioRendercom/x84m583.

We retrieved 14 publications documenting 33 patients (including the present case), of whom 14 were females, 10 were males and 9 without gender report. Age ranges at onset of symptoms were 1-11 months in most patients but 3 cases reported symptoms onset at 20, 27 and 52 years of age. Fourteen patients developed a mycobacterial infection (4 *M. bovis*, 1 *M. avium*, 1 *M. tuberculosis* and the rest did not specify the species of mycobacteria). Among them, 14 distinct PV in the *IKBKB* gene were found. Twenty six cases (including ours) exhibited autosomal recessive inheritance, while the remaining were autosomal dominant. The findings from this review are depicted in **Table 1** and **Figure 3**.

**Table 1:** Variants in the *IKBKB* gene are associated with disease.

Reference	*n*	M/F	Variant (NM/NP) / nationality of origin	Age at debut (months)	Failure to thrive	Hypogammaglobulinemia	Mycobacterial infection	Other infections	Other clinical manifestations and laboratory findings	Hematopoietic stem cell transplant	Death
Index case	1	F	c.1705G>T, (p.Glu569*) homozygous / Mexican	5	–	+	+ *M. bovis* skin, blood, bone marrow	–	Kawasaki disease, hemophagocytic syndrome, acral skin nodules, splenic abscess, pulmonary node	+	+
(3)	1	M	c.1183T>C, (p.Tyr395His) homozygous / Chinese	2	+	+	+	Recurrent respiratory and urinary tract infections and otitis media, severe chronic diarrhea	Reduced production of IL-12 in response to IFN-γ, Low IgG, A, M, low switched-memory B cells, low Tregs, impaired T- and B- cell proliferation	+	–
(4)	4	F	c.1292dupG, (p.Gln432Profs*62) homozygous / Canadian	1	+	–	+ *M. avium*	Parainfluenza virus 1 pneumonia, *E. coli* sepsis, *Pneumococcus* bacteremia, oral candidiasis, osteomyelitis	–	+	+
		F		1.5	+	+	–	*L*. *monocytogenes* sepsis and meningitis, oropharyngeal candidiasis	Small spleen and thymus	–	+
		M		5	+	+	+	Parainfluenza virus 3 pneumonia, oral candidiasis, chronic diarrhea	–	+	–
		F		3	–	+^##^	–	Genitourinary candidiasis, *S.marcescens* sepsis and intracerebral abscess, *E. coli* and *Klebsiella* sepsis, oral candidiasis	–	+	–
(8)	1	F	c.321C>A, (p.Tyr107*) homozygous / Saudi	2	–	+	+ *M. tuberculosis*	Omphalitis, *Salmonella* sepsis, severe recurrent infections caused by *Acinetobacter*, *Enterobacter*, *Stenotrophomonas*, and *Achromobacter* species, rotavirus, and *Candida*. Chronic diarrhea	Delayed separation of the umbilical cord, rash, conical teeth, hepatosplenomegaly, impaired production of IFN-γ, absent production of IL-17, reduced production of TNF-a and IL-6, stimulated IL-12 production reduced and not significantly rescued by addition of exogenous IFN-γ, massive gastrointestinal and pulmonary hemorrhage	–	+
(9)	1	NR	c.736A>G (p.Ser246Gly) homozygous / Brazilian	NR	NR	NR	NR	NR	NR	NR	NR
(10)	1	F	c.814C>T, (p.Arg272*) homozygous / Turkish	3	+	+	+ *M. bovis*	*Pneumocystis jirovecii* pneumonia, *Enterobacter* bacteremia	Progressive respiratory failure, no visible thymus, absence of isotype-switched memory B cells and low CD45R0 memory T cells, conical teeth,arthritis, splenomegaly, rash, ascites	–	+
(11)	1	NR	c.849G>A (p.Trp283*) homozygous / Saudi	NR	+	–	–	Recurrent infections	Two sibling died with the same presentation	NR	NR
(12)	4	F	c.850C>T, (p.Arg284∗) homozygous / Saudi	4	–	+	+	–	Delayed separation of the umbilical cord, rash, pancytopenia hepatosplenomegaly	–	+
		F		3	–	+	–	Perinatal CMV, *Klebsiella pneumoniae* sepsis, urinary tract infections, pneumonia	Delayed separation of the umbilical cord, intracranial calcification chorioretinitis microcephaly, axial hypotonia, hepatosplenomegaly	–	+
		M		3	–	–	+	Oral candidiasis	Delayed separation of the umbilical cord, hepatosplenomegaly, rash	+	–
		M		2	–	–	–	*Klebsiella pneumoniae* sepsis and meningitis	Delayed separation of the umbilical cord	–	+
(13)	4	F	c.856C>T, (p.Arg286*) homozygous / American	5	–	*+*	+	*Candida*	↑CD45RA,CD3 ↑CD45RA,CD3, CD62L	+	–
		M		11	+	+	+	*Candida*, rotavirus, urinary tract infections, Gram negative bacteremia, pneumonias, suppurative otitis media	↓CD45RO,CD3 ↑CD45RA,CD3	+	+
		F		7	+	+	–	*Candida* (oral moniliasis)	↓CD45RO,CD3 ↑CD45RA,CD3	+	+
		M		6	–	+	–	*Candida*, *Klebsiella*, cytomegalovirus *Klebsiella* pneumonia, CMV and oral moniliasis	↓CD45RO,CD3 ↑CD45RA,CD3 ↑CD45RA,CD3, CD62L	+	+
(14)	1	M	c.856C>T; (p.Arg286*) homozygous / Qatari	7	+	IgM↓, IgA↓	+ *M. bovis*	Recurrent respiratory tract infections, recurrent rhinovirus infection, persistent pneumonia, *Streptococcus pneumoniae*, *Klebsiella oxytoca* and *Stenotrophomonas maltophila*, *Burkholderia cepacia complex*	Decreased number of B cells, and decreased percentage NK cells (but normal NK cell number), IgG level was 366 mg/dL (after gammaglobulin infusion), impaired lymphocyte proliferation response to mitogens, decreased spontaneous and antibody-dependent cytotoxicity in NK cells,	+	–
(15)^###^	16	NR	c.1292dupG (p.Gln432Profs*62) homozygous / Canadian	2.5	NR	NR	+	Disseminated CMV (lungs, blood, urine), disseminated Adenovirus (lungs, urine), bacteremia (*Streptococcus pneumoniae*, *Stenotrophomonas maltophilia*)	NR	NR	NR
		NR		1	NR	NR	+	Disseminated CMV (lungs, brain, blood, adrenal glands, heart), *Candida albicans* (oropharyngeal, meningitis), bacteremia (*E. coli*, *Morganella morganni*, *Staphylococcus aureus*) Meningitis (*Staphylococcus aureus*) *Varicella zoster virus*	NR	NR	NR
		NR		3.5	NR	NR	–	Disseminated Adenovirus (nasal, stool, urine), disseminated CMV (Blood), bacteremia (*E. coli*, *Pseudomonas aeruginosa*), thrush	NR	NR	NR
		NR		2	NR	NR	–	Pneumonia (Rhinovirus), *Klebseilla pneumoniae* bacteremia, pneumonia and meningitis, thrush, scabies	NR	NR	NR
		NR		2.5	NR	NR	+ *M. bovis*	Disseminated BCG (meningitis and intracerebral abscesses), fungemia (*Candida albicans*), bacteremia (*Stenotrophomonas maltophilia*, *Enterobacter cloacae*, *E. coli*)	NR	NR	NR
		NR		0	NR	NR	–	–	Newborn screening	NR	NR
		NR		0	NR	NR	–	–	Newborn screening	NR	NR
(16)	1	F	c.1159C>T, (p.Arg446Trp) heterozyygous / English (Caucasian)	20 years	NR	+^##^	NR	NR	Iron deficiency	NR	NR
(17)	1	M	c.512A>G, (p.lLys171Arg) / heterozygous / American	27 years^####^	–	IgG2↑, IgA↓	–	Recurrent otitis media and pneumoniae, cutaneous abscesses	Lymphopenia, Bronchiectasis, progressive pulmonary failure, ectodermal dysplasia, premature cataracts, granulomatous uveitis, hypodontia, and anhidrosis, severe lymphopenia, massive pulmonary hemorrhage	–	+
(18)^#^	4	F	c.607G>A, (p.Val203Ile) heterozygous / Australian, New Zealand and Japanese	NR	–	+	–	Recurrent respiratory tract infections hidradenitis suppurativa, subcutaneous abscesses, mucocutaneous candidiasis	Severe and atypical eczema, dental abnormalities consistent with ectodermal dysplasia (without conical teeth), premature cataracts, defective specific antibody responses to pneumococcal vaccination	NR	NR
		F		NR	–	–	–	Recurrent otitis media and sinusitis	–	NR	NR
		M		NR	–	–	–	Recurrent otitis media and sinusitis	–	NR	NR
		M		NR	–	+	–	Recurrent respiratory infections, otitis media and tonsillitis, subcutaneous abscesses	Bronchiectasis, hepatosplenomegaly	NR	NR
(19)^#^	1	F	c.607G>A; (p.Val203Ile) / heterozygous / German	52 years####	–	IgG2↓, IgG4↓	–	Chronic hepatitis E, herpes zoster infection	Erythemato-telangiectatic rosacea, non-detectable antibodies against pneumococcal vaccine (after sequential conjugated and polysaccharide vaccination), hepatitis B non-responder, significantly reduced CD4^+^and CD8^+^T cells, diminished proportion of CD45RA^+^CD4^+^naïve T cells of 1–2%	–	+

NR, not reported;

^#^The patients were reported with an autosomal dominant inheritance pattern and their clinical manifestations derive from a gain of function of the *IKBKB* gene;

^##^Agammaglobulinemia;

^###^Four patients were previously reported in [4] and they were not included again. Five patients were excluded at this table because they did not have a confirmed genetic diagnosis;

^####^Cases debuted in early childhood but the specific age is not mentioned.

## 4 Discussion

The *IKBKB* gene encodes the IKKβ protein, a serine/threonine kinase that is a key component of the IκB kinase (IKK) complex, critical for activating the NF-κB signaling pathway. The IKKβ protein consists of several domains, each with specific functional roles:

- N-terminal kinase domain: Responsible for the phosphorylation of IκB proteins, leading to their degradation and subsequent activation of NF-κB.
- Helix-loop-helix domain: Involved in protein-protein interactions, particularly within the IKK complex.
- Leucine zipper domain: Facilitates dimerization and interaction with other components of the IKK complex.
- C-terminal NEMO-binding domain (NBD): Essential for binding to NEMO (IKKγ), stabilizing the IKK complex and enabling its activation.

In our patient, the identified variant according to Genome Reference Consortium Human Build 38 (GRCh38) (NM_001556.2) gene: c.1705G>T (p.Glu569*) results in a premature stop codon within the C-terminal region, truncating the protein and likely disrupting the NBD. This truncation would impair the assembly and function of the IKK complex, leading to defective NF-κB signaling and the observed severe immune dysregulation.

*IKBKB* deficiency is a rare immunophenotype characterized by severe combined immunodeficiency (SCID), usually evident in neonates with persistent respiratory or gastrointestinal infections caused by viruses, bacteria, or fungi, often associated with prolonged diarrhea and failure to thrive (3). In contrast, our patient did not present with the typical somatometric involvement seen in neonates. Instead, she debuted later, in infancy, with normal weight and height for her age, initially exhibiting incomplete Kawasaki disease, which later progressed to HLH. SCID was suspected since she came from an endogamous community and *Mycobacterium bovis* was isolated from skin, bone marrow and blood. Also, she had low levels of IgA and IgM and probably IgG, and the flow cytometry reported only that both double-negative T cells and memory B cells were decreased without isotype switching. This is similar to what has already been described in PV of the *IKBKB* gene, since these patients may present normal B-cell and T-cell counts and very low levels of immunoglobulins, as well as a severe defect in immune-cell activation that affects both innate and adaptive immune pathways (4). *IKBKB* deficiency induces abnormal IKKβ protein degradation, leading to impaired NF-kB signaling and immune function. In the absence of stimulatory signals the majority of inactive NF-κB is bound to IkBα and remains in the cell cytosol (3). The BCR and CD40 trigger the canonical NF-kB pathway via activation of the IkB kinase (IKK) complex, which comprises IKKα, IKKβ and NEMO (also known as IKKγ). IKK activation leads to the phosphorylation of IkBα and the subsequent release of the active p50–p65 NF-kB heterodimer, which then translocates to the nucleus to regulate gene transcription. So, impairment of the canonical NF-kB pathway leads to abnormal B cell activation and the patients with hypomorphic variants in *IKBKB* exhibit variable hypogammaglobulinemia (2).

The NF-kB transcription factors are key regulators of inflammatory and immune responses, mediating cell activation, proliferation, survival, and effector functions. The ubiquitously expressed IKK complex links these transcription factors to immune receptors, including T-cell and B-cell receptors, Toll-like receptors, and inflammatory cytokine (TNF- and interleukin [IL]-1ß) receptors (4) and certain variants in the *IKBKB* have been linked to increased susceptibility to autoimmunity and autoinflammation (5, 6). Dysregulation of the NF-κB pathway led to uncontrolled inflammatory responses in our patient, manifesting in conditions such as Kawasaki disease and HLH. In this regards, upon binding to its receptor, IFN-γ activates both the STAT1 and NF-κB pathways, leading to macrophage activation and enhanced IL-12 production, which is essential for eliminating mycobacteria (7). Impairment of the IFN-γ/IL-12 axis predisposes individuals to mycobacterial infections, aligning with previous findings (3).

Our case exhibited hypogammaglobulinemia and a mycobacterial infection, similar to several other cases associated with *IKBKB* PV. However, the isolation of mycobacteria from the skin, blood, and bone marrow in another patient has not been previously reported. Additionally, manifestations of immune dysregulation such as Kawasaki disease and hemophagocytic syndrome have not been documented in connection with this condition.

This case provides valuable insights into the spectrum of alterations in the *IKBKB* gene, including the identification of a novel PV. It also highlights the challenging realities faced by patients undergoing HSCT. Consistent with reports by Cuvelier, et al. (19), our patient developed septic shock just ten days post-transplantation and unfortunately succumbed to it. These findings illustrate the considerable vulnerability of HSCT recipients to severe infections, despite HSCT remaining the only curative treatment currently available.

## 5 Conclusions

In conclusion, we describe a novel homozygous PV in the *IKBKB* gene in our patient, with each parent carrying an affected allele. This finding indicates an autosomal recessive inheritance pattern and expands the mutational spectrum of the gene. Additionally, it confirms the presence of immunodeficiency 15B in our population, revealing clinical manifestations that have not been described until now, such as Kawasaki disease, hemophagocytic syndrome and disseminated tuberculosis affecting skin, blood and bone marrow.

## Data Availability

The datasets presented in this study can be found in online repositories. The names of the repository/repositories and accession number(s) can be found in the article/supplementary material.
